# Data capture, collection, management, and storage systems for clinical research: A scoping review protocol

**DOI:** 10.1371/journal.pone.0346308

**Published:** 2026-09-17

**Authors:** Nicolás Molano-González, Jaime Moreno-Chaparro

**Affiliations:** 1 Clinical Research Group, School of Medicine and Health Sciences, Universidad del Rosario, Bogotá, Colombia; 2 Vice Dean of Research and Consulting, School of Medicine and Health Sciences, Universidad del Rosario, Bogotá, Colombia; Gulu University, UGANDA

## Abstract

The boom and constant transformation of biomedical research have led to the development of different electronic systems or platforms that allow researchers to respond to market demands and clinical research governance. The objective of this scoping review is to map and synthesize existing literature on data capture, collection, management, and storage systems for clinical or hospital research. The methods of this protocol will be used to conduct a scoping review (2000–2025) in accordance with the Joanna Briggs Institute methodological frameworks. Documents that include relevant information about systems for collecting, managing, and storing clinical or hospital research information will be included, following the Population-Concept-Context (PCC) question. Electronic searches will be conducted in Medline (PubMed), EMBASE, SCOPUS, LILACS, Web of Science, and IEEE-Xplore databases, as well as in gray literature through Google Scholar and by snowballing references and citations from studies. The selection and data extraction procedures will be conducted in duplicate and independently. A descriptive narrative synthesis approach to the information will be preferred, with the answers to the research questions being prioritized. This scoping review does not require an ethical review because it does not include the primary information of the participants or the handling of sensitive data. The aim is to identify and analyze relevant literature on clinical research information management systems to provide system developers, clinical researchers, and healthcare institutions with practical frameworks for designing and developing new platforms. This protocol has not completed the search and screening of records.

## Introduction

In recent decades, electronic systems and electronic platforms have been developed for the capture, collection, management, and storage of clinical data, primarily aimed at responding to the growing demand for efficient, qualified, technological, and context-appropriate research services [1,2]. This development is further driven by the increasing need to manage large volumes of data [3] and the importance of focusing on governance and independence in clinical research for organizations [4].

These innovations have transformed current research, contributing significantly to information management and incorporating adjustments and adaptations based on national and international regulations, market and research needs, providing data security, ensuring interoperability between different systems, and improving data quality and analysis [5,6].

Despite these advances and contributions, we still see high variability in the ways and types in which data are collected and managed, as well as a heterogeneous landscape in the origin or source of the data, the use or application of logical and concrete models, the forms of analysis and presentation of information, and the demands on the user or developer [7,8].

Additionally, previous literature on this topic has highlighted the evolution of health data management systems, which were initially identified as not being aligned with the objectives of biomedical care and research (whether due to performance, cost, energy consumption, verification, and use) [9]. Over time, however, these systems have been integrated to create information of sufficient value for research. This remains a challenge worldwide, especially given constant technological change and innovations such as artificial intelligence and natural language [9,10].

A critical gap in current literature lies in how data capture, collection, management, and storage are comprehensively integrated into cohesive electronic ecosystems for clinical research. Existing research frequently examines these components in isolation, focusing heavily on specialized software features rather than evaluating them as interconnected, holistic platforms, with significantly less emphasis placed on dedicated research applications [11]. Furthermore, while prior reviews have addressed health information systems, they predominantly center on clinical care delivery, such as Electronic Health Records (EHRs) [12,13]. The few evaluations that venture into the research domain often restrict their scope to specific medical specialties or traditional Electronic Data Capture (EDC) frameworks [14].

This leaves a notable void in the systematic mapping of versatile, multi-source platforms designed explicitly for rigorous clinical trials and academic research, particularly those incorporating modern capabilities like real-world data integration or high-throughput automated capture. Consequently, a new, comprehensive review is highly necessary to establish a unified, up-to-date landscape of contemporary data systems in clinical research. The rapid acceleration of digital health technologies, coupled with the recent integration of advanced paradigms like artificial intelligence (AI) and decentralized trial designs, has rendered older literature technologically obsolete [15].

In addition to the aforementioned considerations, it is necessary to incorporate a theoretical framework encompassing a digital health systems taxonomy to classify technologies based on their specific operational roles. To operationalize this theoretical taxonomy, contemporary research infrastructures must align with established interoperability frameworks and data standards. Modern EDC platforms no longer operate as isolated data silos; instead, they increasingly rely on Health Level Seven (HL7) and Fast Healthcare Interoperability Resources (FHIR) standards to facilitate automated, eSource-driven data extraction directly from EHRs via secure application programming interfaces (APIs) [16,17].

Based on technological advancement and heterogeneity in the management of clinical data, a comprehensive review is proposed to identify and synthesize the systems-software or electronic platforms focused on the capture, collection, management, and storage of clinical or hospital information for research processes.

Specifically, this review is based on basic concepts such as 1) Variables: the need to collect data within the framework of clinical or hospital research (e.g., sociodemographic information, clinical, diagnostic test results, or laboratory elements, concepts or results of interest, etc.), which in turn respond to predetermined attributes with detailed descriptions and basic validation information [6,18]. 2) Data capture: defined as the process of data entry or acquisition, where information is collected either automatically by devices or manually, and transferred digitally to a study database to optimize entry accuracy [19]. 3) Data collection: focused on the structured accumulation of these data, encompassing the systematic methods and protocols used to gather and compile information from diverse sources [20]. 4) Data management: refers to the governance and processing lifecycle, including the integration, validation, and organization of multiple data types to ensure data integrity and utility for analysis; [21,22] and, finally, 5) Data storage: understood as the underlying infrastructure or database architecture where information is securely housed, remaining centrally accessible, non-volatile, and available for prospective decision-making [6,23].

### Review objectives

This manuscript identifies the following general and specific objectives corresponding to a scope review protocol:

General:

To map and synthesize existing literature on data capture, collection, management, and storage systems for clinical or hospital research.

Specific:

- To identify data capture, collection, management, and storage systems that are focused on clinical research.
- To recognize the architecture with which the identified systems are built.
- To investigate the types of licenses or costs associated with the system use.
- To detail the implementation and operation of the identified systems.

## Methods

### Design

A scoping review will be conducted to answer several questions using various sources of information. At the time of submission of this protocol, the search and screening of records have not been completed. The searches will be conducted in April 2026; subsequently, the record review process is expected to be completed in June 2026. Data extraction is expected to be completed by August 2026. Finally, we expect to have the results of this review in October 2026.

The review will focus on synthesizing available information on clinical research data capture, collection, and storage systems. The methodological framework to be followed is that outlined in the Joanna Briggs Institute manual [24,25], and the Preferred Reporting Items for Systematic Reviews and Meta-Analyses extension for Scoping Reviews (PRISMA-ScR) will be reported [26]. Additionally, this protocol was constructed following the Preferred Reporting Items for Systematic Reviews and Meta-Analyses Protocols (PRISMA-P) checklist (**S1 Table**) [27].

This scoping review will take into account the methodological framework proposed by Arksey and O'Malley [28,29]:

Step 1. A set of research questions was identified.
Step 2. Relevant studies were identified.
Step 3. The studies were selected.
Step 4. Data mapping was conducted.
Step 5. The results were compiled and synthesized
Step 6. Finally, a participatory consultation was conducted with stakeholders or experts.

### Research questions

#### Primary.

What clinical research data capture, collection, management, and storage systems exist?

#### Secondary.

- What is the architecture (software) with which they are built?
- What type of license do they use, and what are the associated costs?
- How does their implementation work in clinical research?
- Are the systems interoperable?

### Selection criteria

According to the selected methodological framework, a strategy based on the identification of population - concept and context (PCC) is sought. Thus, population (P) is understood as all available systems or software; concept (C) is related to the functionality of data capture, collection, management or storage. Finally, context (C) is focused directly on clinical or hospital research.

This search proposes the identification of data from the last 25 years, which corresponds to the boom and growth of relevant literature on the subject. No limitations will be applied in terms of users, geographical region, study designs, and costs.

The exclusion criteria include documents that do not address the topic directly; lack relevant or sufficient information to detail the identified systems; or do not describe or explore the relationship between P-C-C. The selection criteria are summarized in **Table 1**.

**Table 1:** Inclusion and exclusion criteria.

Inclusion	Exclusion
(P) A system, software, or electronic platform.	Studies that do not directly address this topic
(C) Clearly relates to the processes of capturing, collecting, managing, or storing data or information.	Documents that lack relevant information to detail the identified systems or functionalities
(C) Applied in clinical, hospital, or biomedical research.	Do not sufficiently describe or explore the relationship between P-C-C.
Any type or design of study.	Studies that duplicate data or duplicate information related to previous studies
Period 2000–2025.	
Languages: English, Spanish, and Portuguese	

### Search strategy

An advanced search will be conducted in the Medline (PubMed), EMBASE, SCOPUS, LILACS, Web of Science, and IEEE-Xplore databases. The first five databases will be used to collect clinical capture systems, and the last one will be used in technology and engineering research in the same field. The period will be from 2000 to November 2025.

The search for gray literature will focus on identifying documents on Google Scholar’s first five pages. Additionally, potentially relevant articles will be located using the snowball method (searching for references and citations from included studies).

The search strategies will be based on controlled terms (MeSH, EMTREE, and DeCS) and free terms, as well as the use of appropriate Booleans focused on the PCC question. The construction of these search algorithms will be based on the research team’s contributions and the support of a specialized librarian. The strategies will be adjusted according to each database or meta-search engine. **Table 2** lists the main keywords identified and used for each database. The search strategy is outlined in **S2 Table**.

**Table 2:** Relevant keywords for the search.

Population	Data Systems
	Information Systems
Concept	Capture
	Collect
	Management
	Storage
	Warehouse
Context	Clinical
	Biomedical
	Hospital
	Research
	Investigation

* These terms will be combined with Boolean operators (AND – OR) respectively.

### Eligibility process

The information obtained from databases and other sources will be uploaded to a reference management program to eliminate duplicate results. Once this process is completed, the Rayyan QCRI tool will be used to organize the selection process [30]. Titles and abstracts will be evaluated in the first phase, and the full texts of the preliminarily accepted studies will be read in the second phase. This process will be independently carried out by at least two reviewers. In the event of discrepancies between the reviewers’ decisions, an additional (third) reviewer will be brought in to make decisions. For studies that are excluded, the reason will be recorded.

The data extraction process will be independently performed by at least two researchers, following an extraction and cross-verification process. The information will be entered into a predesigned Excel form.

The main data to be extracted are as follows:

1) Bibliographic information (author and year).
2) Manuscript information (place of origin, study design, and main variables); and,
3) System or software information (trade name, date of creation, main objective, system functionality, digital structure or architecture pattern (front and backend), licensing and distribution, costs, interoperability features, implementation processes, and reported outcomes or benefits. The details of the data to be extracted from each study are presented in Table 3.

**Table 3:** Data abstraction approach.

Bibliometrics	Study characteristics	System structure	Outcomes
Author, year of publication, country, journal.	Study design, variables, context, sample size.	Type of system (Capture, Collect, Management, Storage), trade name, date of creation, main objective.	System purpose, digital structure or architecture (front and backend), licenses or availability costs, interoperability features, implementation processes (barriers and facilitators), and main outcomes or benefits.

### Methodological quality assessment

The quality of the included studies will not be assessed according to the methodological frameworks described, as the nature of scoping reviews and their objective is based on identifying and synthesizing as much information on a given topic as possible [24].

### Data analysis

The included studies, as well as the basic and specific information to be collected in the extraction phase, will be summarized using a quantitative (counts, frequencies, and percentages) and qualitative descriptions (nominal and detailed).

To explore the complex structural intersections between software characteristics, advanced data visualizations will be generated. Specifically, Sankey diagrams will be utilized to illustrate the flow and organizational relationships between system architectures, operational domains, and deployment targets. Additionally, weighted co-occurrence matrices will be mapped to analyze the integration density and configuration alignment among different system functionalities.

#### Classification taxonomy and system grouping.

A predefined coding framework will be developed to systematically classify and analyze the characteristics of digital systems. The coding framework will include the following domains:

1. System functionality and purpose. Systems will be grouped by their core function into EDC, Clinical Trial Management Systems (CTMS), Data Warehouses, or Hybrid/Integrated platforms.
2. Architectural pattern. The technical deployment architecture will be classified into Cloud-based, On-premise, or Hybrid architectures.
3. Licensing and Distribution. Systems will be distinguished by their legal and business model into Open-source or Proprietary/Commercial licensing.
4. Interoperability features. Variables will include integration with EHRs, laboratories, registries, or external databases, as well as the use of APIs or other interoperability standards.
5. Implementation characteristics. Include the implementation setting, required expertise, training needs, barriers, facilitators, and other reported challenges or experiences.
6. Cost and licensing model. System is open-source, proprietary, or mixed, along with any reported costs for acquisition, maintenance, or implementation.
7. Reported outcomes or benefits. May include improvements in data quality, efficiency, workflow, collaboration, compliance, user satisfaction, or research productivity.

#### Qualitative thematic analysis and coding framework.

- Cross-study exploration and conceptual mapping: A matrix will be constructed to contrast technical strategies across different settings. This will enable the identification of recurring rationales behind architectural choices, convergence, and divergence across domains.
- Triangulation and synthesis integration: Quantitative trends (e.g., the prevalence of cloud architectures) will be systematically triangulated with qualitative themes (e.g., specific security or cost challenges) to synthesize cohesive findings and identify structural gaps in the current literature.

Where sufficient data are available, subgroup analyses will be conducted to explore differences according to system type, architecture, licensing model, implementation context, and reported costs.

### Patient and public involvement

As part of the development of a good scoping review [29], it is expected that a call for dissemination of the results will be made prior to publication, including, above all, experts in the field of engineering as well as expert clinical researchers. Previously, experts in the field sought opinions to obtain information that would enable the development of this protocol. Finally, experts in the areas of systems and health seek to complement the vision of this review and contribute appropriately to a high-quality academic product (in terms of clarity, quality, and application of findings).

## Discussion

Given the increasing volume of data and the need to strengthen data governance and organizational independence in clinical research, electronic platforms for data capture, collection, management, and storage have become key infrastructure for optimizing workflow efficiency and ensuring data integrity [3,4].

Therefore, understanding the different existing systems, detailing them, and having access to the information will help optimize information management, ensure compliance with national and international regulations, guarantee interoperability between systems, and improve data quality and analysis.

The strengths of this review are related to conducting advanced searches in databases, gray literature, and the snowball method to find relevant information. This review protocol was developed according to the protocol development reporting items. The scoping review will follow the methodological framework of the Joanna Briggs Institute and will report the items for scoping reviews in their extended version [24,25].

Limitations may relate to the number and management of documents, as multiple clinical use systems can be reported without going into detail. Additionally, due to the nature of this type of review and the recommendations of the methodological framework, the methodological quality of the documents found will not be evaluated. This limits the results, as no statements will be made about the strength or validity of the evidence.

Furthermore, this scoping review is inherently constrained by publication bias, industry secrecy, and rapid technological evolution. Because the clinical research software market is heavily dominated by commercial, closed-source platforms that rarely disclose their internal architectures due to intellectual property restrictions, a systematic underreporting of proprietary systems exists, potentially biasing the findings toward open-source frameworks. Additionally, the accelerated pace of digital health innovation (driven by advancements in AI, decentralized methodologies, and shifting interoperability protocols) introduces a distinct technological evolution bias. Since academic publication lifecycles require fixed administrative timelines, certain evaluated software systems or features may undergo substantial updates or obsolescence by the time of publication. Consequently, the compiled evidence must be interpreted as a static, temporal snapshot of a highly dynamic technological landscape.

This scoping review will not include the collection of sensitive patient or institution data. Additionally, it focuses solely on documents that mention systems or software focused on the collection, management, and storage of data for the management of clinical research and therefore does not require specific ethical approval.

## Conclusion

This scope review protocol presents a detailed step-by-step guide for conducting a scope review focused on mapping and synthesizing existing literature on data capture, collection, management, and storage systems for clinical or hospital research. This information may be valuable to researchers, research centers, and hospitals that wish to use these systems for clinical research.

Dr. NMG is the guarantor of the scoping review protocol.

## Supporting information

S1 Table PRISMA-P-checklist. (DOCX)

S2 Table Search algorithm preliminary. (DOCX)
