# Supplementary material for: Data capture, collection, management, and storage systems for clinical research: A scoping review protocol
Source: PLoS One. 2026 Sep 17;21(9):e0346308. doi: 10.1371/journal.pone.0346308 (PMC13585225; doi:10.1371/journal.pone.0346308)
Supplement: Supplementary file 2 [file pone.0346308.s002.docx]

**S2 Table.** Search algorithm preliminary

Search

**#1** Systems[Title/Abstract]

**#2** Software[MeSH Terms]

**#3** #1 OR #2

**#4** Data[Title/Abstract]

**#5** Capture[Title/Abstract]

**#6** Collect[Title/Abstract]

**#7** Management[Title/Abstract]

**#8** Storage[Title/Abstract]

**#9** #5 OR #6 OR #7 OR #8

**#10** #4 AND #9

**#11** Clinical[Title/Abstract]

**#12** Hospital[Title/Abstract]

**#13** Biomedical[Title/Abstract]

**#14** #11 OR #12 OR #13

**#15** Research[Title/Abstract]

**#16** #14 AND #15

**#17** #10 AND #16

**#18** #3 AND #17

**#19** #18 AND 2000-2025
